# Combined Ultrahigh Pressure Extraction and High-Speed Counter-Current Chromatography for Separation and Purification of Three Glycoside Compounds from *Dendrobium officinale* Protocorm

**DOI:** 10.3390/molecules26133934

**Published:** 2021-06-28

**Authors:** Wei Zhang, Yingjie Zhang, Jinying Wang, Wenjuan Duan, Feng Liu

**Affiliations:** 1 School of Pharmaceutical Sciences, Shandong Academy of Sciences, Qilu University of Technology, Jinan 250014, China; zhangwei01101212@163.com (W.Z.); jewel_wong@foxmail.com (J.W.); duanwj4048@126.com (W.D.); 2 Shandong Analysis and Test Center, Key Laboratory for Applied Technology of Sophisticated Analytical Instruments of Shandong Province, Shandong Academy of Sciences, Qilu University of Technology, Jinan 250014, China; trudysunny@163.com

**Keywords:** ultrahigh pressure extraction (UPE), high-speed counter-current chromatography (HSCCC), PLBs of *D. officinale*, glycoside compounds

## Abstract

As an alternative to *Dendrobium candidum*, protocorm-like bodies (PLBs) of *Dendrobium candidum* are of great value due to their high yield and low cost. In this work, three glycoside compounds, β-D-glucopyranose 1-[(E)-3-(4-hydroxyphenyl)-2-propenoat] (I), β-D-glucopyranose 1-[(E)-3-(3, 4-dihydroxyphenyl)-2-propenoat] (II), and 1-O-sinapoyl glucopyranoside (III), were extracted and isolated by ultrahigh pressure extraction (UPE) coupled with high-speed counter-current chromatography (HSCCC) from PLBs of *D. officinale*. First, the target compounds were optimized and prepared with 50% ethanol solution at a 1:30 (g/mL) solid/liquid ratio in 2 min under 300 MPa by UPE. Then, the crude extract was chromatographed with a silica gel column, and primary separation products were obtained. In addition, the products (150 mg) were separated by HSCCC under the solvent system of MTBE-*n*-butyl alcohol-acetonitrile-water (5:1:2:6, *v*/*v*/*v*/*v*), yielding 31.43 mg of compound I, 10.21 mg of compound II, and 24.75 mg of compound III. Their structures were further identified by ESI-MS, ^1^H NMR, and ^13^C NMR. The antioxidant results showed that the three compounds expressed moderate effects on the DPPH· scavenging effect. Compound II had the best antioxidant capacity and its IC_50_ value was 0.0497 mg/mL.

## 1. Introduction

A culture of protocorm-like bodies (PLBs) of *Dendrobium candidum* has been obtained by using a tissue culture to induce the regeneration of *D. officinale* [1], which has the advantages of easy planting, short growth cycle, and low cost [2]. Its unique medical value and health benefits have led to the excessive use of *D. officinale*, which has a low reproduction rate in nature, and the resources of *D. officinale* are in short supply [3,4,5]. The PLBs are considered as an excellent material to replace *D. officinale* [6]. PLBs of *D. officinale* have the activities of treating skin problems and strengthening the immune system, and they have antioxidant, anti-tumor, anti-cancer, and anti-inflammatory activities [7,8,9]. At present, the research of PLBs of *D. officinale* has mainly focused on cultivation [10,11], long-term preservation [12], gene expression [13,14], etc., and there are few reports on the active components in PLBs [15,16], among which the separation of glycosides from PLBs of *D. officinale* has not been reported.

Glycoside compounds are derivatives of polysaccharides and play an important role in the treatment of liver diseases [16] and diabetes [17], and they have anti-tumor [18] and neuroprotective effects [19,20]. However, the separation of these compounds has always been a major problem in natural medicinal chemistry research because of their highly polarity-sensitive property. Most of their separation has mainly been with column chromatography [21,22], which has obvious disadvantages such as high time-consumption and solvent pollution, and low yield and efficiency. Thus, it is unsuitable for the large-scale production of glycoside compounds in this traditional way [23,24]. In this regard, an effective and ecofriendly approach for isolating glycosides from PLBs of *D. officinale* is required.

Ultrahigh pressure extraction (UPE) subjects the material to extraction pressures between 100 and 800 MPa, which can increase the mass transfer level and accelerate the diffusion of active components. In recent years, it has been used to extract active compounds from natural products due to its advantages of low energy consumption, shortened extraction time, high efficiency, etc. [25,26]. Compared with conventional column chromatography, high-speed counter-current chromatography (HSCCC) is a continuous and efficient liquid-liquid extractive chromatography with no solid support [27,28,29], which can also eliminate the irreversible adsorption problem of the sample and separate different substances from the two phases through different distribution coefficients [30,31]. HSCCC has the advantages of recoverable samples, less solvent consumption, and low cost [32,33]. A combined application of UPE and HSCCC could be a good choice for an effective extraction and separation method.

**Figure 1:**
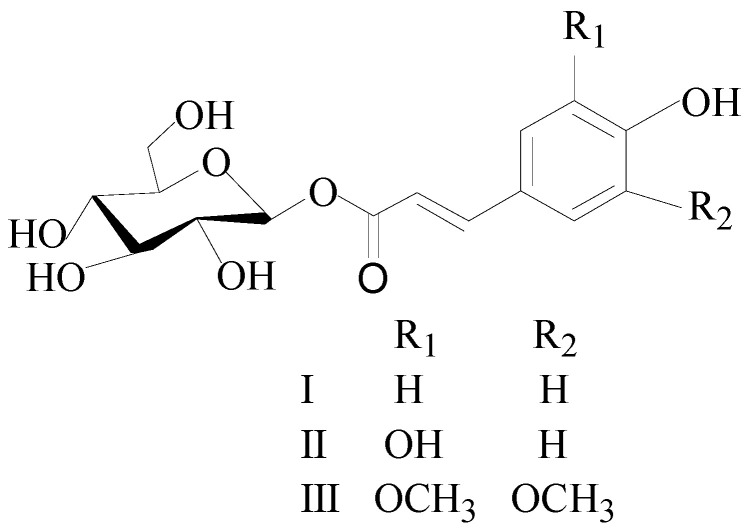
The chemical structure of the three compounds. I: β-D-glucopyranose 1-[(E)-3-(4-hydroxyphenyl)-2-propenoat]; II: β-D-glucopyranose 1-[(E)-3-(3, 4-dihydroxyphenyl)-2-propenoat]; III: 1-O-sinapoyl glucopyranoside.

In this study, an extraction and purified method of three glycoside compounds, β-D-glucopyranose 1-[(E)-3-(4-hydroxyphenyl)-2-propenoat] (I), β-D-glucopyranose 1-[(E)-3-(3, 4-dihydroxyphenyl)-2-propenoat] (II), and 1-O-sinapoyl glucopyranoside (III) (Figure 1), from the PLBs of *D. officinale* was successfully established by a combination of UPE and HSCCC.

## 2. Results and Discussion

### 2.1. Ultrahigh Pressure Extraction Parameters

The evidence shows that the factors affecting the UPE may include pressure level, pressure holding time, type and concentration of solvent, time to achieve treatment pressure, decompression time, product initial temperature, and product pH [34]. To investigate the apparent parameters for UPE, we optimized four of them by changing the concentrations of extraction solvents, the extraction pressure, the extraction time, and the liquid/solid ratio in the action system. The yield of the target compounds was used as the marker for extraction efficiency evaluation.

#### 2.1.1. The Effect of Ethanol Concentration

**Figure 2:**
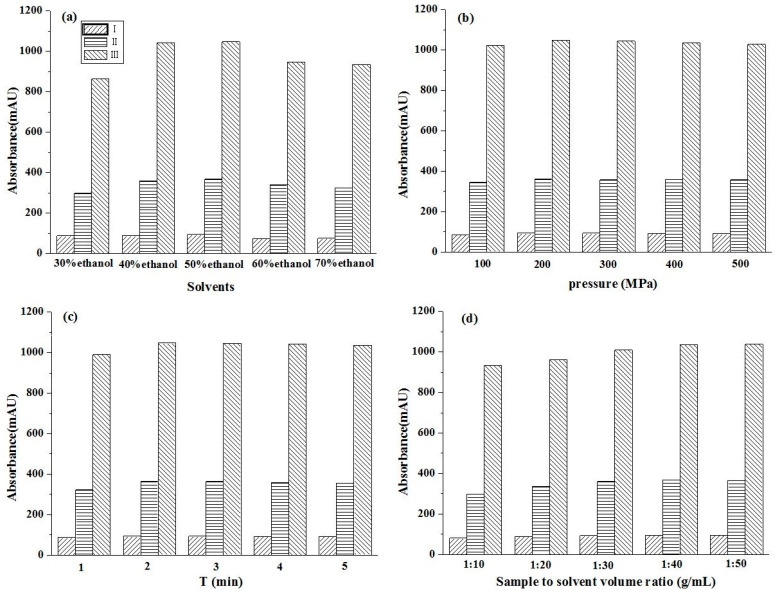
Effects of solvents (**a**), extraction pressure (**b**), extraction time (**c**), and sample solvent volume ratio (g/mL) (**d**) on the contents of target compounds by UPE. (I) β-D-glucopyranose 1-[(E)-3-(4-hydroxyphenyl)-2-propenoat]; (II) β-D-glucopyranose 1-[(E)-3-(3, 4-dihydroxyphenyl)-2-propenoat]; (III) 1-O-sinapoyl glucopyranoside.

The concentration of the extraction solvent will affect the solubility of the target component, and different solvents will produce different target compounds. To assess this, five different concentrations of aq. ethanol were used as solvents, and UPE extraction was performed at 300 MPa, 2 min, and with a 1:20 (g/mL) solid/liquid ratio. The yields of glycoside compounds extracted with different concentrations of aq. ethanol are shown in Figure 2a. When the aq. ethanol concentration ranged from 30% to 50%, the increased glycoside extraction rate was observed with an increasing aq. ethanol concentration. In contrast, the extraction rate of glycosides decreased as the aq. ethanol concentration increased from 50% to 70%. The extraction rate of the three glycoside compounds reached its highest with the aq. ethanol concentration of 50%. The polarity of the extraction solvent was similar to that of the target compound. Besides, ethanol is the most commonly used nontoxic organic solvent as the extraction solvent. Therefore, in the further experiments, 50% ethanol solution was chosen for the following experiments.

#### 2.1.2. The Effect of Pressure

Ultrahigh pressure can increase the mass transfer rate and the solubility of glycosides, which is directly associated with the extraction rate and extraction time. The effect of pressure was investigated by extracting samples at pressures from 100 to 500 MPa with other parameters such as a 50% ethanol solution, 2 min, and a 1:20 (g/mL) solid/liquid ratio. As shown in Figure 2b, the extraction yield increased as the pressure rose from 100 to 300 MPa. Meanwhile, no obvious change was observed when the pressure was in the range of 300–500 MPa. Therefore, the pressure of 300 MPa was selected for subsequent experiments.

#### 2.1.3. The Effect of Extraction Time

The effect of extraction time was investigated in the range from 1 to 5 min with other conditions such as 300 MPa, 50% ethanol solution, and a 1:20 (g/mL) solid/liquid ratio, and the results are shown in Figure 2c. The extraction yield increased with extraction time from 1 to 2 min. However, there was no obvious increase in their extraction yields with the extraction times of 3, 4, and 5 min. This was because the different pressures between the inner and outer cell membranes were large enough to cause instant permeation and to obtain the highest yield rapidly under high pressure [35]. Therefore, the extraction time of 2 min was selected.

#### 2.1.4. The Effect of Solid-Liquid Ratio

As presented in Figure 2d, a clear relationship between the yield of the three compounds and the solid-liquid ratio was revealed with a 50% ethanol solution under a 300 MPa pressure for 2 min. Gradually increased yields were observed with the decrease in the solid-liquid ratio. Taking the solvent consumption and processing cost into consideration, the solid-liquid ratio of 1:30 (g/mL) was chosen as the best ratio in subsequent experiments.

According to the results of single factor experiments, the suitable conditions of the three target compounds extracted by UPE were 50% ethanol solution, 300 MPa extraction pressure, 2 min extraction time, and 1:30 (g/mL) solid-liquid ratio. Using the above UPE conditions, the extraction yields of three compounds were 2.84, 5.89, and 15.31 mg/g, respectively.

### 2.2. Comparison of UPE and Hot Reflux Extraction

The extract yields of three glycoside compounds from PLBs implemented by UPE and traditional heat reflux extraction were compared. Under the conditions of Section 3.4, the extraction yields of the three compounds by HRE were 2.71, 4.18, and 15.23 mg/g, respectively. The results showed that UPE attained slightly higher extraction yields than those of HRE, while reducing the extraction time from 60 to 2 min and not requiring heating. Therefore, UPE was an effective and rapid technique for the extraction of the three glycosides from PLBs.

### 2.3. Selection of the Optimized Two-Phase Solvent System

In order to successfully obtain monomeric compounds through HSCCC, the *K_D_* value of each target compound should be considered. Generally, the *K_D_* value for the target component is usually expected to be between 0.5 and 2.0. If the *K_D_* value is under 0.5, the solute will be eluted near the solvent front, which may result in peak resolution loss and poor separation performance; if the *K_D_* value is much greater than 2.0, the solute will elute in an excessively broad peak and may lead to a longer elution time, reduced column efficiency, and wasted solvent [36,37,38]. Meanwhile, the stationary phase retention can reflect the exaction rate during HSCCC separation. The *K_D_* values of three compounds in different solvent systems are shown in Table 1.

First, a chloroform-methanol-water (4:3:3, *v*/*v*/*v*) two-phase solvent system was selected according to the properties of the target compounds. However, the *K_D_* value was too large, the target compound eluted for a long time, and the resolution was poor. Then, a series of highly polar solvent systems with varying volume ratios composed of MTBE-*n*-butanol-acetonitrile-water were used to efficiently resolve the compounds.

It can be seen from Table 1 that when the MTBE-*n*-butanol-acetonitrile-water (4:2:3:8, *v*/*v*/*v*/*v*) solvent system was used, the *K_D_* values of the three compounds were 0.39, 0.79, and 0.95, respectively, which was in line with the relatively ideal range of *K_D_* values. The *K_D_* values of the three compounds were also greater than 0.5 and less than 2 in the MTBE-*n*-butanol-acetonitrile-water (5:1:2:6, *v*/*v*/*v*/*v*) two-phase solvent system. Therefore, these two solvent systems were suitable for the target compounds separation.

**Figure 3:**
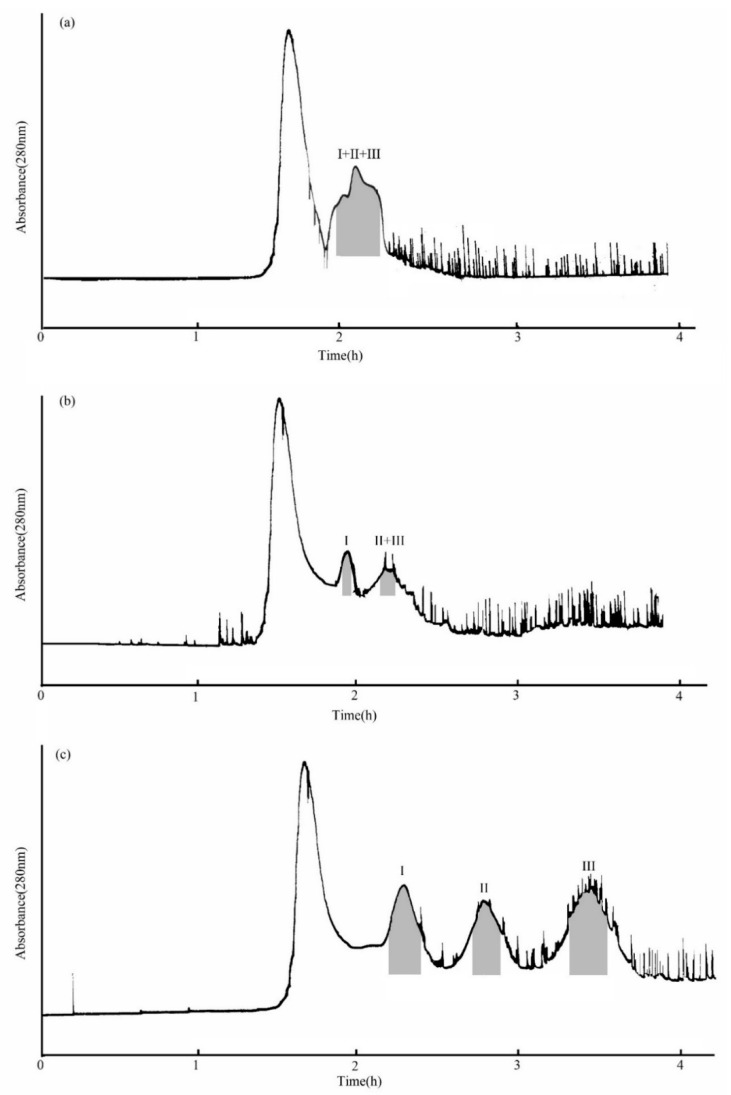
HSCCC chromatogram of the crude sample from PLBs of *D. officinale*. (**a**) MTBE-*n*-butyl alcohol-acetonitrile-water (4:2:3:8, *v*/*v*/*v*/*v*) system, retention of stationary phase: 36.7%, flow rate: 2.0 mL/min; (**b**) MTBE-*n*-butyl alcohol-acetonitrile-water (5:1:2:6, *v*/*v*/*v*/*v*) system, retention of stationary phase: 35.9%, flow rate: 2.0 mL/min; (**c**) MTBE-*n*-butyl alcohol-acetonitrile-water (5:1:2:6, *v*/*v*/*v*/*v*) system, retention of stationary phase: 41.67%, flow rate: 1.0 mL/min. Peak I: β-D-glucopyranose 1-[(E)-3-(4-hydroxyphenyl)-2-propenoat], Peak II: β-D-glucopyranose 1-[(E)-3-(3,4-dihydroxyphenyl)-2-propenoat], Peak III: 1-O-sinapoyl glucopyranoside.

The MTBE-n-butanol-acetonitrile-water (4:2:3:8, *v*/*v*/*v*/*v*) two-phase solvent system for the separation solvent system was used first. As Figure 3a shows, when the flow rate of the aqueous phase was 2.0 mL/min, the three compounds eluted together. Based on this, the MTBE-n-butanol-acetonitrile-water (5:1:2:6, *v*/*v*/*v*/*v*) two-phase solvent system was used as the separation solvent for the second time. The evidence showed that although this system provided a suitable *K_D_* value at a 2.0 mL/min flow rate of the aqueous phase, stationary phase retention was small (about 35.9%). In addition, compound I was separated, but compounds II and III were eluted together (Figure 3b) under this condition. The reason may be that the flow rate was too fast to separate compounds II and III. Therefore, the flow rate was adjusted to 1.0 mL/min, and the others remained unchanged in the next experiment. The results indicated that the stationary phase retention was 41.67%, and compounds I, II, and III were successfully isolated (Figure 3c). The elution flow rate decreased with the length of the separation column, which means it improved the separation effect.

### 2.4. Purification of Three Compounds by HSCCC

**Figure 4:**
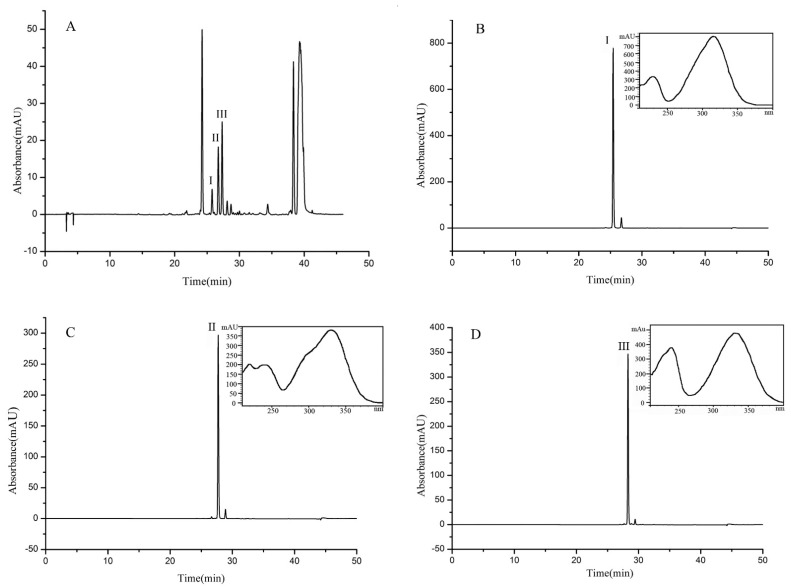
HPLC chromatogram of the extract from UPE (**A**) and HPLC chromatograms and UV spectra of compounds I (**B**), II (**C**), and III (**D**) separated and purified by HSCCC.

A 150 mg sample (fraction 4, separated as Section 3.5) was separated and purified in the MTBE-*n*-butyl alcohol-acetonitrile-water (5:1:2:6, *v*/*v*) solvent system. As shown in Figure 3c, three compounds were separated in one step by HSCCC in 4 h. Finally, 31.43 mg of β-D-glucopyranose 1-[(E)-3-(4-hydroxyphenyl)-2-propenoat] (I) with a purity of 97.8%, 10.21 mg of β-D-glucopyranose 1-[(E)-3-(3, 4-dihydroxyphenyl)-2-propenoat] (II) with a purity of 98.6%, and 24.75 mg of 1-O-sinapoyl glucopyranoside (III) with a purity of 98.4% were obtained, the purities being determined by HPLC, as shown in Figure 4.

### 2.5. Identification of Purified Compounds

The structures of the purified compounds were identified by the comparison of their ESI-MS, ^1^H NMR, and ^13^C-NMR data with those in the literature (see Supplementary Materials).

Compound I: positive ESI-MS *m*/*z*: 349[M+Na]^+^ C_15_H_18_O_8_, ^1^H NMR (400 MHz, DMSO) δ:7.62 (1H, d, J = 16 Hz, HC-7), 7.56 (2H, d, J = 8 Hz, HC-2, 6), 6.79 (2H, d, J = 8Hz, HC-3, 5), 6.37 (1H, d, J = 16 Hz, HC-8), 5.44 (1H, d,J = 8Hz,HC-1’). 13C-NMR (400 MHz, DMSO): 165.8(s, C-9), 160.6 (s, C-4), 146.4(d, C-7), 131.0 (d, C-2, 6), 125.4 (s, C-1), 115.4 (d, C-3, 5), 114.0 (d, C-8), 94.7 (d, C-1’), 78.3 (d, C-3’ or 5’), 76.9 (d, C-5’ or 3’), 72.8 (d, C-2’), 69.9 (d, C-4’), 61.0 (t, C-6’). The data were in accordance with a previous report [39], and compound I was identified as β-D-glucopyranose 1-[(E)-3-(4-hydroxyphenyl)-2-propenoat].

Compound II: positive ESI-MS *m*/*z*: 365[M+Na]^+^. C_15_H_18_O_9_, ^1^H NMR(400 MHz, DMSO) δ:7.54 (1H, d, J = 16Hz, HC-7), 7.07 (1H, d, J = 2 Hz, HC-2), 7.00 (1H, dd, J = 8 Hz and 2Hz, HC-6), 6.77 (iH, d, J = 8 Hz, HC-5), 6.25 (1H, d, J =16Hz, HC-8), 5.43 (1H, d, J = 8 Hz, HC-1’).^13^C-NMR (400 MHz, DMSO) δ:165.8 (s, C-9), 150.1 (s, C-4), 148.4(d, C-7), 146.7 (s, C-3), 125.8 (s, C-i), 123.8 (d, C-6), 116.1 (d, C-2), 114.4 (d, C-8), 111.8 (d, C-5), 94.7 (d, C-i’), 78.3 (d, C-3’ or 5’), 77.0 (d, C-5’ or 3’), 73.0 (d, C-2’), 70.0 (d, C-4’), 61.1 (t, C-6’). The data were in agreement with the literature [40], and compound II was defined as β-D-glucopyranose 1-[(E)-3-(3, 4-dihydroxyphenyl)-2-propenoat].

Compound III: positive ESI-MS *m*/*z*: 409[M+Na]^+^. ^1^H NMR(400 MHz, DMSO) δ: 7.05(2H, s, H-2 and H-6), 7.64 (1H, d, J = 15.9 HZ, H-7), 6.55(1H, d, J = 15.9 HZ, H-8), 3.80 (6H, s, OCH_3_), 5.46 (1H, d, J = 8.0 HZ, H-1’); ^13^C-NMR (400 MHz, DMSO) δ: 124.75(C-1), 106.88(C-2 and C-6), 148.52(C-3 and C-5), 147.08 146.02 (C-4), 139.07(C-7), 114.82(C-8), 165.87(C-9), 56.57 (CH_3_), 94.70(C-1’), 73.04(C-2’), 78.31(C-3’), 70.03(C-4’), 77.01(C-5’), 61.10(C-6’). The data of compound III were consistent with those of 1-O-sinapoyl glucopyranoside reported in reference [40].

### 2.6. DPPH Radical Scavenging Effect

**Figure 5:**
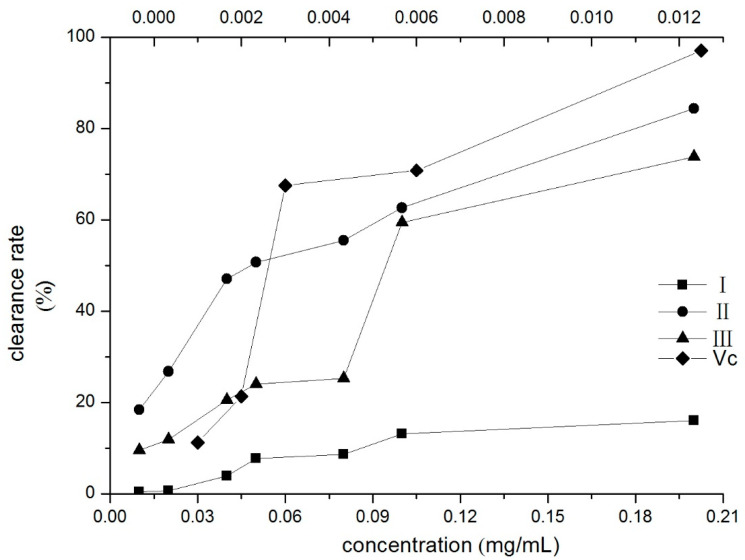
Scavenging effects of three compounds on DPPH assay (the upper horizontal axis was Vc’s). I: β-D-glucopyranose 1-[(E)-3-(4-hydroxyphenyl)-2-propenoat], II: β-D-glucopyranose 1-[(E)-3-(3, 4-dihydroxyphenyl)-2-propenoat], III: 1-O-sinapoylglucopyranoside.

To verify the antioxidant activity of compounds I–III, DPPH radical scavenging effect tests were performed. The DPPH radical scavenging ability of the three compounds increased with the concentration, as shown in Figure 5. The IC_50_ value indicates the concentration of the corresponding compound when the scavenging rate reaches 50% [41]. The IC_50_ values of compounds II and III were 0.0497 and 0.1111 mg/mL, respectively. Compound II had the best DPPH radical scavenging activities in three compounds.

## 3. Experimental Section

### 3.1. Reagents

Organic solvents, including TMBE, n-butyl alcohol, and acetonitrile for HSCCC, were all purchased from Juye Chemical Factory (Jinan, China). Acetonitrile for HPLC analysis was obtained from Yuwang Chemical Factory (Yucheng, China). All reagents were at analytical class without further purification. The water used in solutions and dilutions was treated with a Milli-Q water purification system (Millipore, Burlington, MA, USA). Ultrapure water was prepared by the Milli-Q water purification system (Millipore, Burlington, MA, USA) for solutions and dilutions.

### 3.2. Apparatus

The ultrahigh pressure-assisted extraction was performed with an HPP.L3-600 High Hydrostatic Pressure Processor (Huataisenmiao Biology Engineering Technology Co. Ltd., Tianjin, China). The pressure ranged from 0 to 900 MPa, and the pressure precision was ±5 MPa.

The separation of HSCCC was carried out by a TBE-300A apparatus (Tauto Biotech, Shanghai, China) with a series of three multilayer coil columns (2.6 mm i.d. total capacity of 300 mL) and a 20 mL sample loop. The above system was also equipped with a Model TBP1002 constant-flow pump (Tauto Biotech, Shanghai, China), a Model 8823B-UV detector (Beijing Tianchen Biotech Co., Ltd., Beijing, China), and a DCW-0506 circulatory temperature regulator (Shanghai Baidian Instrument Co., Ltd., Shanghai, China). A Model STR1001 portable recorder (Jiangsu shun tong instrument Co., Ltd., Xuzhou, China) was used to record the chromatogram.

HPLC analysis was performed with Agilent 1120 HPLC equipment (Agilent, Santa Clara, CA, USA) using a YMC-Pack ODS-A column (5 µm, 250 mm × 4.6 mm, i.d.). ESI-MS and NMR spectra analysis and identification were performed on an Agilent 6520 Q-TOF (Agilent, Santa Clara, CA, USA) and a Bruker AV-400 spectrometer (Bruker BioSpin, Rheinstetten, Germany), respectively.

### 3.3. UPE Conditions

The dried PLBs of *D. officinale* powder (1 g) was mixed with a given number of solvents, and it was then moved into a sterile polyethylene bag. The bag was sealed after the removal of bubbles, and then the mixture was subjected to UPE treatment for a given period. The different ethanol solution concentrations (30%, 40%, 50%, 60%, and 70% ethanol solution, *v*/*v*), extraction pressures (100, 200, 300, 400, and 500 MPa), extraction times (1, 2, 3, 4, and 5 min), and solid-liquid ratios (1:10, 1:20, 1:30, 1:40, and 1:50 g/mL) were studied. The extracting solution was centrifuged at 4000 rpm for 10 min, and the supernatant was filtered through a 0.45 μm membrane. Then, the filtrate was injected into the HPLC for further analysis. After the UPE conditions were optimized, 150 g of sample powder was extracted.

### 3.4. Heat Reflux Extraction

Here, 120 mL of 50% (*v*/*v*) methanol solution as the extraction solvent was added to 10 g of dried plant powder. The temperature and time of the heat reflux extraction were fixed at 75 °C and 1 h, respectively.

### 3.5. Silica Gel Column Chromatography Separation

The crude product (63g) extracted by UPE was purified by column chromatography on silica gel using petroleum:ether-ethyl acetate (4:1-1:1, *v*/*v*) as the eluent solvent. The eluted fraction was combined together according to TLC analysis. Fraction 1 was obtained by eluting petroleum:ether-ethyl acetate (4:1, *v*/*v*), and fractions 2, 3, and 4 were eluted and achieved by the petroleum:ether-ethyl acetate (3:1, 2:1, and 1:1) solvent, respectively. Fraction 4 (13.6 g) was further purified by HSCCC.

### 3.6. Selection of Two-Phase Solvent System and Preparation of Sample for HSCCC

The separation success with HSCCC largely relies on the selection of a suitable two-phase solvent system, which provides an ideal range of partition coefficients (*K_D_*) for the target compounds. First, after configuring all the two-phase solvent systems, the appropriate amount of sample that needs to be separated by HSCCC was dissolved in the upper and lower phases of the same volume. When the distribution equilibrium was attained, the upper and lower phases were respectively percolated through a 0.45 μm membrane filter, and the same solution was taken for analysis. The *K_D_* value was calculated using following formula [36]:

*K_D_* = *C_U_*/*C_L_*

where *K_D_* is the partition coefficient, *C_U_* is the concentration of the target compound in the upper phase, and *C_L_* is the concentration of the target compound in the lower phase.

Then, based on the target compound partition coefficient (*K_D_*) and stationary phase retention, two-phase HSCCC solvent systems were selected, namely chloroform-methanol-water (4:3:3, *v*/*v*/*v*), MTBE-*n*-butanol-acetonitrile-water (4:2:3:8, *v*/*v*/*v*/*v*), MTBE-*n*-butanol-acetonitrile-water (5:1:2:6, *v*/*v*/*v*/*v*), MTBE-*n*-butanol-acetonitrile-water (2:0:2:3, *v*/*v*/*v*/*v*), and MTBE-*n*-butanol-acetonitrile-water (6:0:3:8, *v*/*v*/*v*/*v*).

### 3.7. HSCCC Separation Procedures

For the HSCCC experiment, the two-phase solvent system of TMBE-*n*-butyl alcohol-acetonitrile-water (5:1:2:6, *v*/*v*/*v*/*v*) was placed in a separator funnel for use. The HSCCC separation procedures were as follows: the solvent system was separated in a separatory funnel after equilibrium, and the upper and lower phases were degassed with an ultrasound bath for 15 min before use. First, the upper phase was pumped into the multilayer coiled column and entirely filled at a flow rate of 15 mL/min; then, the lower aqueous phase was pumped into the column at a flow rate of 2.0 mL/min using the head to end mode at 800 rpm. After hydrodynamic equilibrium was established, the sample solution containing 150 mg of sample (fraction 4, separated as Section 3.5) was injected into the separation column through the injection loop. The effluent collected manually every 6 min was monitored online using a UV detector at 280 nm at a flow rate of 1.0 mL/min. When the HSCCC separation was finished, the retention rate of the stationary phase was calculated as the volume retained in the stationary phase divided by the total volume of the column.

### 3.8. HPLC Analysis and Identification of HSCCC Fractions

The crude sample was first isolated with a silica column. Then, the purified fractions separated via HSCCC were analyzed by HPLC at room temperature. The mobile phases of a solution of acetonitrile (A) and 0.2% formic acid (B) in gradient mode were as follows: 0–5 min: 5% A, 5–15 min: 5–10% A, 15–25 min: 10–15% A, 25–35 min: 15–20% A, 35–40 min: 20–25% A, 40–41 min: 25–100% A, 41–50 min: 100–100% A; the flow-rate was set at 1 mL/min. The effluent was monitored by a PDA at 280 nm.

The identification of the HSCCC peak fractions was performed by ESI-MS on an Agilent 1100/MS-G1946, and NMR spectra were recorded on a Varian-600 spectrometer (Varian, Palo Alto, CA, USA) with tetramethylsilane (TMS) as an internal standard.

### 3.9. DPPH Radical Scavenging Effect

According to the method in [41], the antioxidant capacity of the three target compounds was evaluated with minor modifications. Compounds I–III were serially diluted at concentrations of 0.01, 0.02, 0.04, 0.05, 0.08, 0.1, and 0.2 mg/mL in ethanol, which were used as sample solutions. DPPH· ethanol solution (3 mL, 0.08 mmol/L) was added to the test tubes, which had 2 mL of sample solution, and it was then allowed to react for 30 min under dark conditions at room temperature. The absorbance value of every group was determined under 517 nm by ultraviolet spectrophotometry. The IC_50_ values were calculated by dose-scavenging curves using Origin 8.5 software (version 8.5, OriginLab, Northampton, MA, USA).

## 4. Conclusions

In this study, a significantly efficient method for the extraction and purification of β-D-glucopyranose 1-[(E)-3-(4-hydroxyphenyl)-2-propenoat], β-D-glucopyranose1-[(E)-3-(3, 4-dihydroxyphenyl)-2-propenoat], and 1-O-sinapoyl glucopyranoside from PLBs of *D. officinale* was achieved by UPE coupled with HSCCC. The suitable conditions for UPE of the target compounds were 50% ethanol, 300 MPa of pressure, 2 min of extraction time, and a 1:30 (g/mL) solid/liquid ratio. The crude extraction of UPE was chromatographed over a silica gel column and eluted with a solvent system composed of petroleum:ether-ethyl acetate (1:1, *v*/*v*). The eluting fraction was successfully purified by HSCCC using a MTBE-*n*-butyl alcohol-acetonitrile-water (5:1:2:6, *v*/*v*/*v*/*v*) solvent system. Particularly, this method revealed a good performance in separating and purifying glycosides compounds with similar polarity, which may provide a reference for the rapid extraction and effective isolation of these substances in the future.

## Figures and Tables

**Table 1:** The *K_D_* values of three compounds in different solvent systems.

Solvent System	Volume Ratio (*v*/*v*)	*K_D_* Values		
		Compound I	Compound II	Compound III
MTBE-*n*-butyl alcohol-acetonitrile-water	4:2:3:8	0.39	0.79	0.95
	5:1:2:6	0.98	1.24	1.45
	2:0:2:3	0.23	0.36	0.39
	6:0:3:8	0.21	0.38	0.43
chloroform-methanol-water	4:3:3	2.56	2.98	3.79

## Data Availability

The data presented in this study are available on request from the corresponding author.

## Supplementary Materials

The following are available online, Figures S1–S3: ^1^H-^13^C-NMR spectra of compound Ⅰ; Figures S4–S7: ^1^H-^13^C-NMR spectra of compound Ⅱ; Figures S8–S10: ^1^H-^13^C-NMR spectra of compound Ⅲ.

## Sample Availability

Not available.
