# Supplementary material for: Combined Ultrahigh Pressure Extraction and High-Speed Counter-Current Chromatography for Separation and Purification of Three Glycoside Compounds from Dendrobium officinale Protocorm
Source: Molecules. 2021 Jun 28;26(13):3934. doi: 10.3390/molecules26133934 (PMC8271780; doi:10.3390/molecules26133934)
Supplement: Supplementary file 1 [file molecules-26-03934-s001.zip › molecules-1200353 - supplementary materials-v2.pdf]

Supplementary Materials

Combined Ultrahigh Pressure Extraction and High-Speed Counter-Current Chromatography for Separation and Purification Three Glycoside Compounds from *Dendrobium officinale* Protocorm

Wei Zhang [1,2], Yingjie Zhang [2], Jinying Wang [1,2], Wenjuan Duan [1,2] and Feng Liu [1,2,*]

[1] School of Pharmaceutical Sciences, Qilu University of Technology (Shandong Academy of Sciences), Jinan, 250014, China; zhangwei01101212@163.com (W.Z.); jewel_wong@foxmail.com (J.W.); duanwj4048@126.com (W.D.)

[2] Shandong Analysis and Test Center, Key Laboratory for Applied Technology of Sophisticated Analytical Instruments of Shandong Province, Qilu University of Technology (Shandong Academy of Sciences), Jinan, 250014, China; trudysunny@163.com

* Correspondence: liufeng8109@qlu.edu.cn or liufeng8109@163.com ; Tel.: +86-0531-82605319

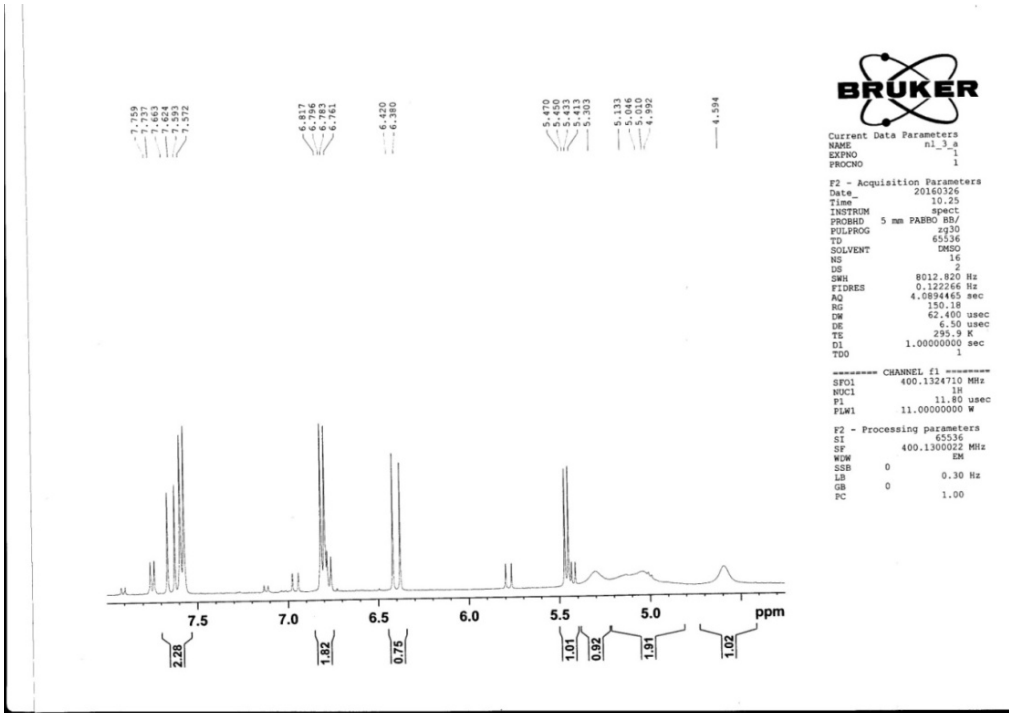

Figure S1. ^{1}H NMR of compound I（β-D-glucopyranose1-[(E)-3-(4-hydroxyphenyl)-2-propenoat]）.

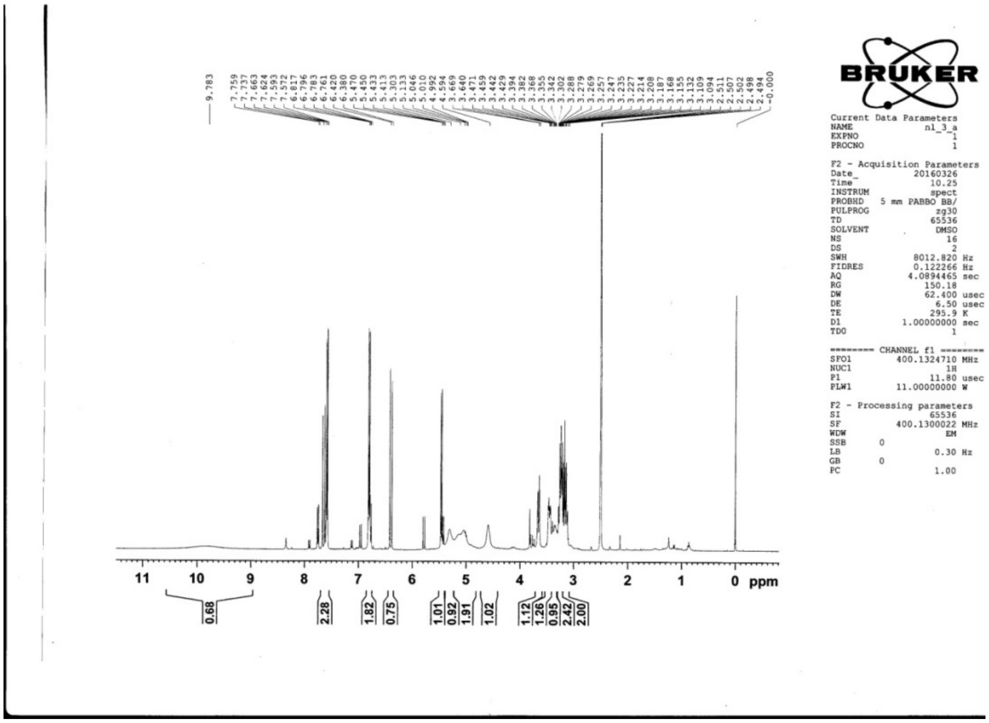

Figure S2. ^{1}H NMR of compound I（β-D-glucopyranose1-[(E)-3-(4-hydroxyphenyl)-2-propenoat]）.

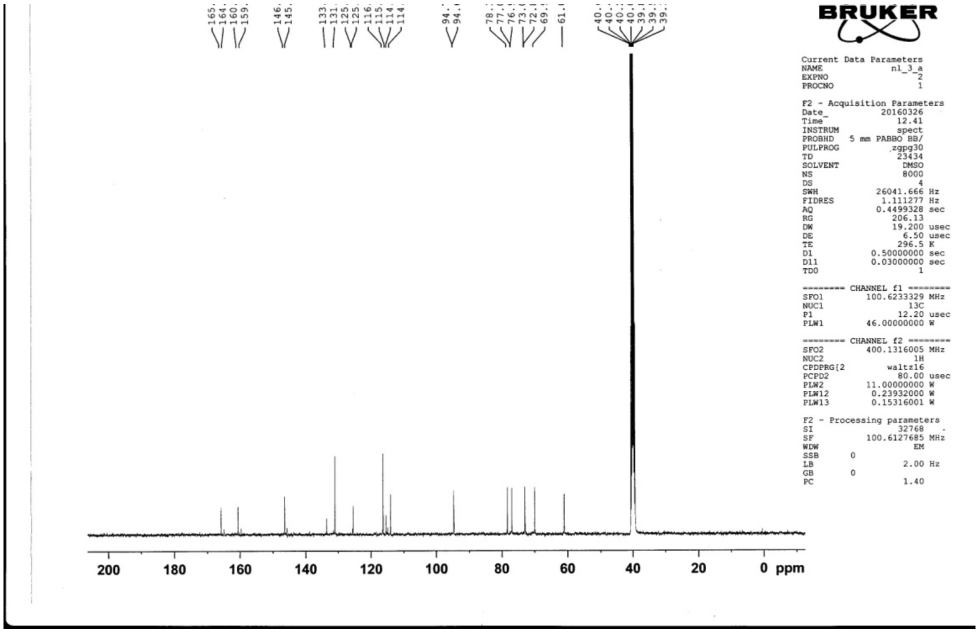

Figure S3. ^{13}C NMR of compound I（β-D-glucopyranose1-[(E)-3-(4-hydroxyphenyl)-2-propenoat]）.

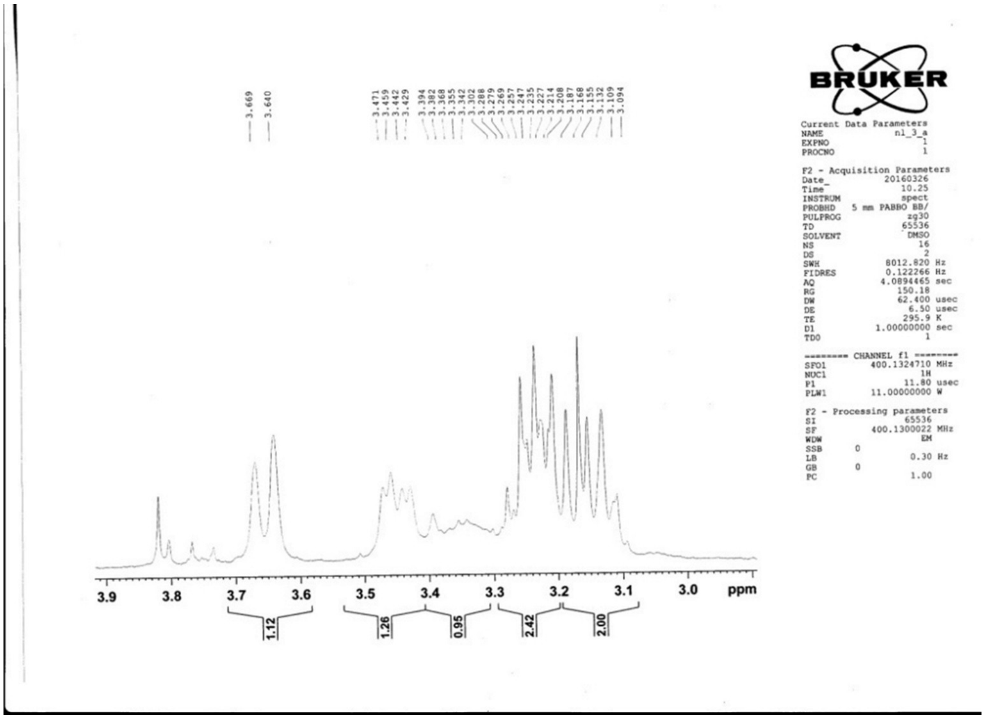

Figure S4. ¹H NMR of compound II（β-D-glucopyranose 1-[(E)-3-(3, 4-dihydroxyphenyl)-2-propenoat]）.

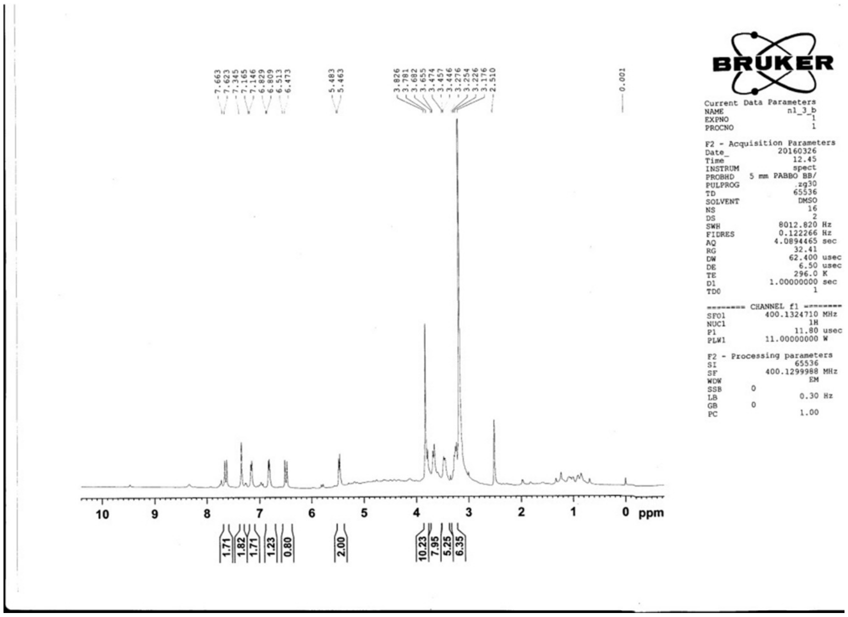

Figure S5. ¹H NMR of compound II（β-D-glucopyranose 1-[(E)-3-(3, 4-dihydroxyphenyl)-2-propenoat]）.

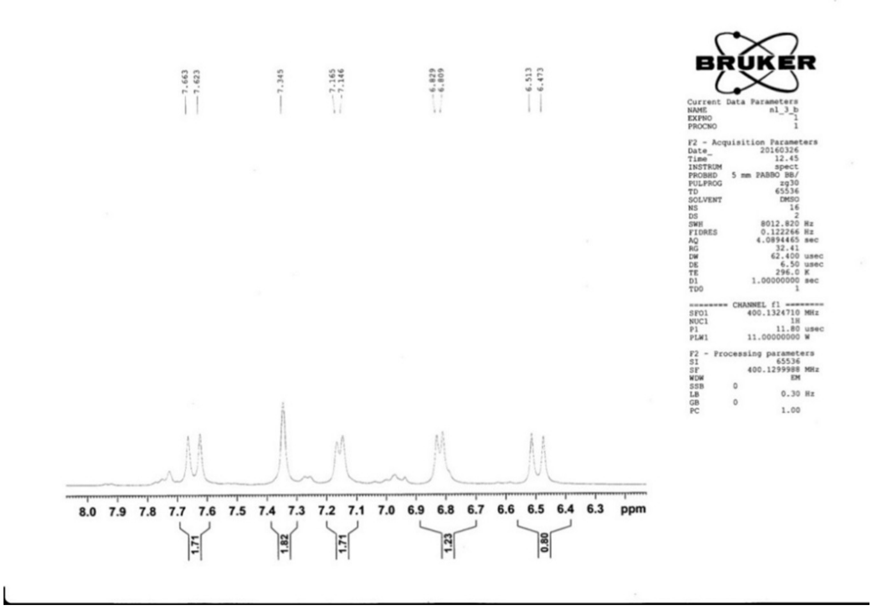

Figure S6. [13]H NMR of compound II（β-D-glucopyranose 1-[(E)-3-(3, 4-dihydroxyphenyl)-2-propenoat]）.

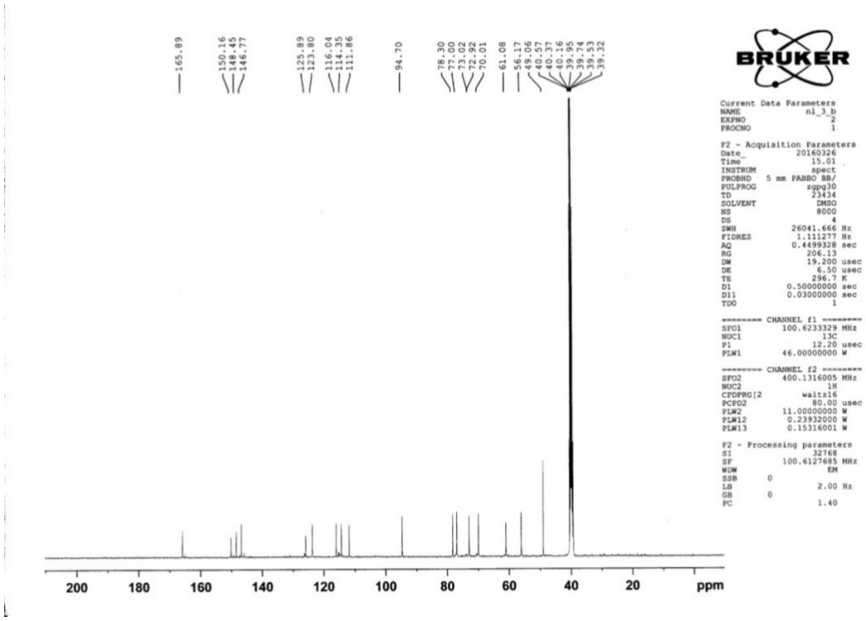

Figure S7. ^{13}C NMR of compound II（β-D-glucopyranose 1-[(E)-3-(3, 4-dihydroxyphenyl)-2-propenoat]）.

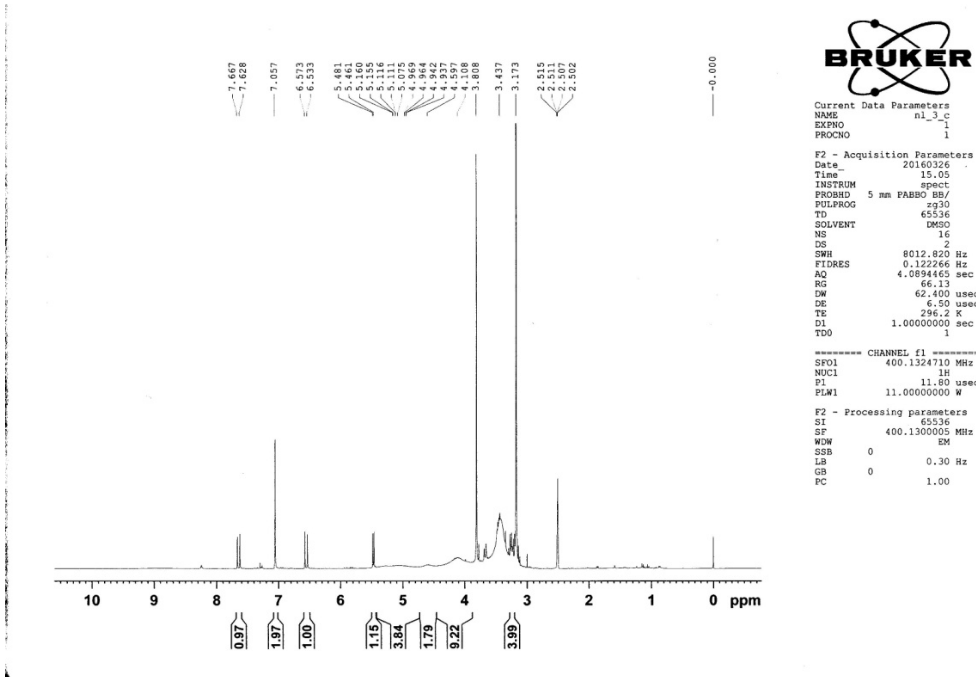

Figure S8. ^{13}H NMR of compound III（1-O-sinapoyl glucopyranoside）.

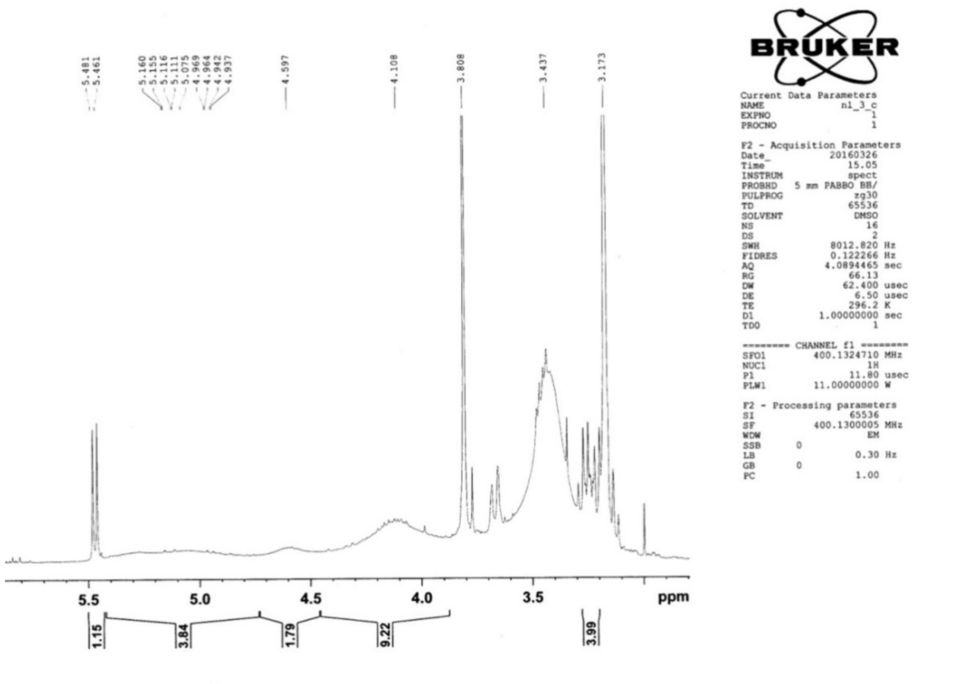

Figure S9. ^{13}H NMR of compound III（1-O-sinapoyl glucopyranoside）.

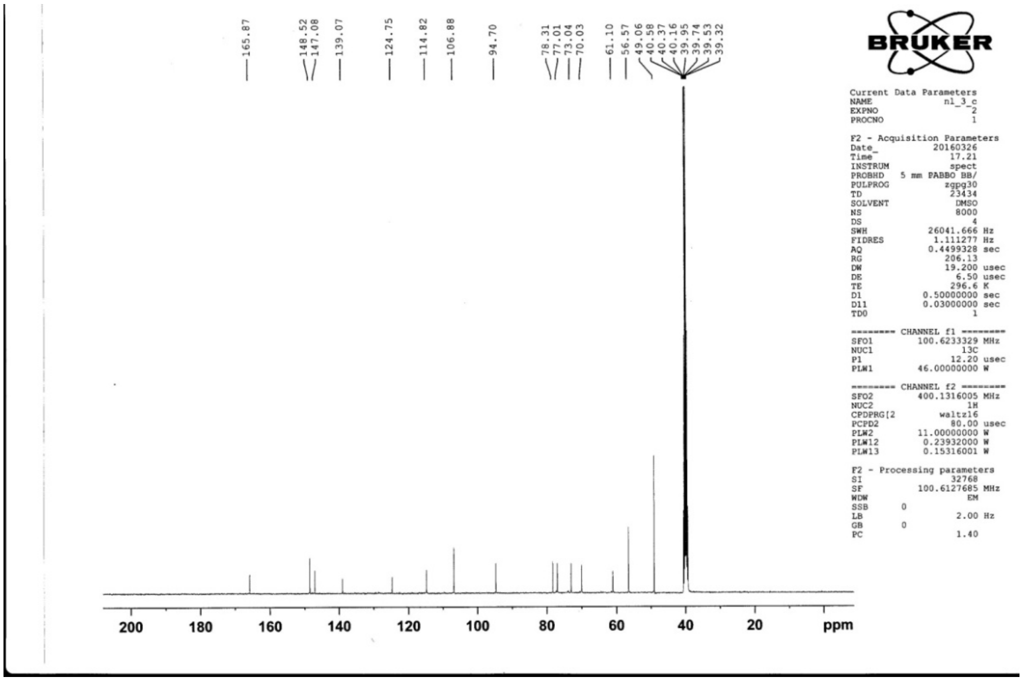

Figure S10. ^{13}C NMR of compound III（1-O-sinapoyl glucopyranoside）.
